# Mortality Statistics, Chicago

**Published:** 1875-09

**Authors:** Ben. C. Miller

**Affiliations:** Sanitary Superintendent


***Chicago Mortality Report for July, 1875.*** Reported by Dr. Ben. C. Miller, Sanitary Superintendent.

Board of Health of the City of Chicago,
Health Office, Chicago, Aug. 11, 1875.

*To the Editors of the Chicago Medical Journal and Examiner :*

Sirs—I enclose you the mortality report for July. You will see that compared with June, the increase is 638, but compared with the corresponding month of last year, shows a decrease of 283. This makes a good showing for the sanitary condition of Chicago. The mean thermometer has been lower than last year, but subjected to greater variations; this has been accompanied by an unusual number of cases of bowel affections, but fortunately of not a fatal character. If we get through the summer with as good a comparison with the past as we have up to date, we will have reason to congratulate ourselves. Respectfully yours,

BEN. C. MILLER, *San. Supt.*

---

| | |
|---|---|
| Accident, burns | 1 |
| " crushed | 2 |
| " drowning | 14 |
| " fall | 2 |
| " run over by 'bus | 1 |
| " scalding | 2 |
| " railroad | 5 |
| " shooting | 2 |
| " strangulation | 1 |
| Abscess, psoas | 1 |
| Abscesses | 2 |
| Anæmia | 2 |
| Apoplexy | 6 |
| Arachnitis | 1 |
| Bowels, consumption of | 2 |
| " obstruction of | 1 |
| " ulceration of | 1 |
| Brain, abscess of | 1 |
| " congestion of | 12 |
| " disease of | 1 |
| " inflammation of | 2 |
| " softening of | 4 |
| Bronchitis | 10 |
| " capillary | 3 |
| Cancer | 1 |
| " of arm | 1 |
| " of breast | 3 |
| " of face | 1 |
| " of liver | 2 |
| " of neck | 1 |
| " of rectum | 1 |
| " of stomach | 2 |
| " of uterus | 3 |
| Cholera infantum | 412 |
| " morbus | 12 |
| Consumption | 42 |
| Convulsions | 146 |
| " puerperal | 2 |
| Croup | 14 |
| Cyanosis | 1 |
| Cystitis | 3 |
| Debility, general | 8 |
| Diabetes | 1 |
| Diarrhœa | 80 |
| " chronic | 4 |
| Diphtheria | 7 |
| Dropsy | 3 |
| Dysentery | 28 |
| Endo-carditis | 2 |
| Enteritis | 23 |
| Entero-colitis | 24 |
| Erysipelas | 2 |
| Fever, congestive | 2 |
| " intermittent | 1 |
| " puerperal | 4 |
| " remittent | 1 |
| " scarlet | 11 |
| " typhoid | 12 |
| Gastritis | 3 |
| Gastro-enteritis | 6 |
| Hæmorrhage | 1 |
| Heart, disease of | 4 |
| " dropsy of | 1 |
| " hypertrophy of | 1 |
| " mitral valves, disease of | 1 |
| " rheumatism of | 1 |
| " embolism of | 2 |
| " rupture of | 1 |
| " valvular disease of | 3 |
| Hepatitis | 1 |
| Hip joint, disease of | 1 |
| Hernia | 1 |
| Hydrocephalus | 9 |
| Inanition | 29 |
| Jaundice | 1 |
| Kidneys, Bright's disease of | 3 |
| " disease of | 4 |
| Liver, atrophy of | 1 |
| " cirrhosis of | 2 |
| Lungs, congestion of | 4 |
| " emphysema of | 1 |
| Malformation | 1 |
| Manslaughter | 2 |
| Metro-peritonitis | 1 |
| Measles | 11 |
| Meningitis | 19 |
| " cerebro-spinal | 8 |
| " tubercular | 6 |
| Old age | 12 |
| Paralysis | 2 |
| Pericarditis | 2 |
| Peritonitis | 3 |
| Pneumonia | 19 |
| " typhoid | 1 |
| Pyæmia | 2 |
| Rheumatism | 1 |
| Spina bifida | 1 |
| Stomach, congestion of | 1 |
| " ulceration of | 3 |
| Suicide | 3 |
| Syphilis | 3 |
| Tabes mesenterica | 16 |
| Teething | 12 |
| Trismus | 1 |

| | |
|---|---|
| Uræmia | 2 |
| Urine, retention of | 1 |
| Uterus, sarcoma of | 1 |
| Whooping cough | 11 |
| Total | 1,171 |

Premature births, 9; Still births, 65. Total, 74.

COMPARISON.

Deaths in July, 1875, 1,171; in June, 1875, 533. Increase, 638. Deaths in July, 1874, 1454. Decrease, 283.

AGES.

| | |
|---|---|
| Under one year | 671 |
| One year to two | 204 |
| Two years to three | 31 |
| Three " " four | 17 |
| Four " " five | 14 |
| Five " " ten | 17 |
| Ten " " twenty | 36 |
| Twenty " " thirty | 40 |
| Thirty years to forty | 45 |
| Forty " " fifty | 24 |
| Fifty " " sixty | 26 |
| Sixty " " seventy | 22 |
| Seventy " " eighty | 19 |
| Eighty " " ninety | 5 |
| Total | 1,171 |

| | | | | | |
|---|---|---|---|---|---|
| White | 1,160 | Males | 629 | Married | 146 |
| Colored | 11 | Females | 542 | Single | 1,025 |
| Total | 1,161 | Total | 1,171 | Total | 1,171 |

NATIVITIES.

| | | | | | |
|---|---|---|---|---|---|
| Belgium | 1 | England | 14 | Scotland | 2 |
| Bohemia | 4 | France | 1 | Sweden | 4 |
| Canada | 4 | Germany | 65 | Wales | 1 |
| Native—Chicago | 225 | Holland | 2 | Unknown | 8 |
| Foreign, " | 679 | Ireland | 42 | | |
| U. States, other parts | 111 | Norway | 5 | Total | 1,171 |
| Denmark | 3 | | | | |

Deaths daily, 37¾. Mean thermometer, 68.7°. Rain fall, 7.20 inches.

MORTALITY BY WARDS.

| Wards. | No. Deaths. | Pop. in 1874. | Percentage. | Wards. | No. Deaths. | Pop. in 1874. | Percentage. |
|---|---|---|---|---|---|---|---|
| 1 | 4 | 5,725 | one death in 1,431 | 11 | 30 | 14,022 | one death in 467 |
| 2 | 4 | 4,830 | " " 1,208 | 12 | 24 | 16,792 | " " 699 |
| 3 | 20 | 14,861 | " " 743 | 13 | 21 | 17,892 | " " 852 |
| 4 | 20 | 15,361 | " " 768 | 14 | 33 | 16,720 | " " 507 |
| 5 | 51 | 20,078 | " " 394 | 15 | 237 | 45,545 | " " 192 |
| 6 | 151 | 35,916 | " " 238 | 16 | 78 | 21,922 | " " 281 |
| 7 | 112 | 31,722 | " " 283 | 17 | 49 | 20,777 | " " 424 |
| 8 | 77 | 29,143 | " " 379 | 18 | 54 | 21,392 | " " 394 |
| 9 | 80 | 31,654 | " " 398 | 19 | 9 | 4,677 | " " 519 |
| 10 | 18 | 17,385 | " " 966 | 20 | 15 | 8,995 | " " 599 |

Ratio of deaths to population in 1874, one death in 338.

| | |
|---|---|
| No. deaths in Wards | 1,087 |
| Accidents | 30 |
| County Hospital | 6 |
| Foundlings' Home | 28 |
| Hospital Alexian Bros. | 6 |
| Home for Friendless | 1 |
| Good Shephards | 1 |
| Mercy Hospital | 2 |
| Manslaughter | 2 |
| Protestant Orphan Asylum | 1 |
| St. Joseph's Hospital | 2 |
| Women's Refuge | 2 |
| Suicides | 3 |
| Total | 1,171 |

